# Oncology Recapitulates Surgical Anatomy: Re-Defining Negative Oncologic Margins with R1 Vascular Hepatectomy in Colorectal Liver Metastases

**DOI:** 10.3390/cancers18142188

**Published:** 2026-07-08

**Authors:** Corey A. Hounschell, Jared A. Forrester, Ronald F. Wolf, Anton Bilchik, Paul Shin

**Affiliations:** 1 Division of Surgical Oncology, Saint John’s Cancer Institute, Santa Monica, CA 90404, USA; corey.hounschell@providence.org (C.A.H.);; 2 Division of Surgical Oncology, University of California at San Diego, San Diego, CA 92182, USA; jforrester@health.ucsd.edu; 3 Division of Transplantation and Hepatobiliary Surgery, University of California at San Diego, San Diego, CA 92182, USA; 4 Division of Hepatopancreaticobiliary Surgery, Hoag Memorial Presbyterian Hospital, Newport Beach, CA 92663, USA; ronald.wolf@hoag.org

**Keywords:** colorectal liver metastasis, R1 vascular margin, parenchyma-sparing surgery, Laennec’s capsule, precision oncology

## Abstract

Complete resection with microscopically negative (R0, or no tumor cells within 1 mm of resection surface) margins has long been considered the cornerstone of curative-intent hepatectomy for colorectal liver metastases (CRLM). R1 resection is defined as the presence of tumor cells at or within 1 mm of the inked margin. A growing body of contemporary evidence, however, shows that the traditional binary R0/R1 framework obscures biologically meaningful subcategories of positive margin. An R1 parenchymal (R1p) margin on the transected liver surface carries a distinctly adverse prognosis, whereas an R1 vascular (R1v) margin (created by intentional detachment of tumor from the adventitia of a major intrahepatic vessel along Laennec’s capsule) yields oncologic outcomes that are, in essence, equivalent to R0: modern series report five-year overall survival rates of 34–45% after R1v, comparable to matched R0 cohorts. This insight has radical implication, enabling parenchyma-preserving hepatectomy (PPH) for patients who would otherwise require major hepatectomy (i.e., trisectionectomy) for multifocal and centrally located tumors, as well as allowing patients who would otherwise be considered unresectable to undergo curative intent resection. Careful patient selection, taking into consideration anatomic variation, use of intraoperative ultrasound guidance, response to systemic therapy, and molecular tumor profiling is essential for the safe application of R1v PPH. This review synthesizes the evidence supporting the R1p/R1v distinction, places it within the broader evolution of margin paradigms in CRLM, and outlines the clinical, anatomical, and biological criteria that should guide its judicious use in the current era of precision hepatobiliary surgery.

## Simple Summary

For patients undergoing liver resection for colorectal liver metastases (CRLM), early surgical pioneers in the 1980s believed that a wide margin of healthy liver tissue around the tumor was required to maximize survival benefit. Over time, new insights into segmental liver anatomy and long-term follow up data from parenchymal preserving hepatectomy (PPH) for CRLM have progressively challenged this precept, re-defining adequate margins in terms of “no tumor on ink.” Most recently, new clinical and histopathologic insights have challenged the very idea that the optimal margin in oncologic liver surgery is per se defined by negative margins.

## 1. Introduction

For most of the modern history of hepatic surgery, complete resection with microscopically negative margins (R0) has been regarded as the sine qua non of curative-intent surgery for colorectal liver metastases (CRLM). Early retrospective experience, most influentially that of Fong and colleagues in their analysis of 1001 consecutive hepatic resections for metastatic colorectal cancer, established a framework in which margin status was a central prognostic variable alongside tumor number, size, nodal status of the primary tumor, and carcinoembryonic antigen level [1]. Within the framework, R0 denotes complete microscopic clearance (≥1 mm tumor-free margin), whereas R1 indicates microscopic positivity at the inked edge. An R1 margin was treated as a surrogate for oncologic failure, and the subsequently derived “1-cm rule” dominated surgical decision-making for more than two decades.

A pivotal multi-institutional analysis by Pawlik and colleagues was the first to challenge this rule. In their series, a positive margin remained strongly associated with worse overall survival, yet the width of the negative margin did not significantly influence survival. The authors concluded that complete R0 resection, rather than the number of millimeters of clearance, was the critical determinant of outcome [2]. This finding was later corroborated by a propensity-matched analysis of more than 2700 patients from two high-volume United Kingdom centers. In that study, a 1 mm cancer-free margin was sufficient to achieve 33% five-year disease-free survival, and additional margin width conferred no further disease-free survival advantage [3].

Since the publication of these earlier studies, several convergent developments have prompted a more granular reinterpretation of margin status in CRLM. Propensity-matched and multivariable analyses have consistently shown that R1 status is tightly correlated with adverse tumor biology and disease burden. As a result, unadjusted survival differences between R0 and R1 cohorts largely disappear once these confounders are taken into account [4,5,6]. Against this backdrop, a major conceptual advance has emerged through the prospective subclassification of R1 resections according to anatomical location. An R1 parenchymal (R1p) margin is defined by the presence of tumor cells within 1 mm of the transected liver surface. In contrast, an R1 vascular (R1v) margin occurs when tumor is deliberately detached from the adventitial surface of a major intrahepatic vessel along Laennec’s capsule [7,8]. These two entities behave very differently, with contemporary series demonstrating that R1v yields survival rates often indistinguishable from R0 in comparable populations [7,9]. This review discusses the anatomic and biologic rationale for this distinction, along with its evolution from earlier margin paradigms, and delineates the clinical, anatomical, and biological criteria that should guide the selective, intentional use of R1v in modern hepatobiliary practice.

## 2. Evolution of the Margin Paradigm in Colorectal Liver Metastases

The conceptual journey from the 1-cm rule to the modern R1p/R1v distinction has unfolded in three overlapping phases. Each phase has been shaped by advances in surgical technology, pathology, and systemic therapy (Table 1).

### 2.1. Challenging the 1-cm Rule

Through the 1990s, surgical dogma held that a resection margin of at least 10 mm was required for a curative hepatectomy for CRLM. This view was systematically eroded by several large series in the early 2000s. Pawlik and colleagues demonstrated that once an R0 margin was achieved, additional clearance beyond 1 mm conferred no meaningful survival advantage [2]. Subsequent pathological studies established that intrahepatic micrometastases around CRLM are uncommon, and, when present, they are almost always confined to within a few millimeters of the tumor edge [10]. These observations provided a biological rationale for why sub-centimeter margins were oncologically sufficient. In a propensity-matched analysis of 2715 patients, Hamady and colleagues established that a 1 mm margin was sufficient for curative-intent resection, with no additional disease-free survival benefit from wider clearance [3]. This 1 mm threshold is now the widely accepted modern definition of an R1 resection [14,15].

### 2.2. Understanding the Heterogeneous Biology of "Close Margins"

As the 1 mm threshold became standard, attention shifted from the millimeters of clearance toward the biology of the patients in whom close margins occurred. In a cohort of 273 patients, Truant and colleagues reported that although patients undergoing R1 resection exhibited inferior five-year overall, disease-free, and progression-free survival on univariate analysis, this difference did not hold after multivariable adjustment [6]. A propensity score-matched analysis by Sakai and colleagues showed similar results. Apparent differences in recurrence-free and overall survival between R0 and R1 patients disappeared once baseline tumor burden was balanced. The authors concluded that R1 resection itself is not a cause of poor prognosis but rather a potent indicator of aggressive tumor biology [5]. In the largest single-center series to date, Sadot and colleagues analyzed 2368 patients and identified a stepwise survival gradient across margin widths (0 mm, 0.1–0.9 mm, 1–9 mm, ≥10 mm). Even submillimeter clearance conferred a significant survival advantage over a 0 mm margin. The authors argued that margin width was most plausibly a microscopic surrogate for the biologic behavior of a tumor rather than the result of surgical technique [11].

Parallel lines of evidence have clarified the role of systemic therapy in modulating the significance of margins. Ayez and colleagues demonstrated that the survival detriment associated with R1 resection largely disappeared in patients who received neoadjuvant chemotherapy [16]. Hosokawa and colleagues showed that long-term cure remained achievable after R1 resection, (5-year OS 48%, with cure in 18%), with limited cycles of preoperative chemotherapy and objective response as independent predictors of cure [17]. Tanaka and colleagues found that the R0–R1 survival gap was abolished in patients with initially unresectable disease who responded to chemotherapy [18]. Laurent and colleagues similarly showed that R1 resection did not independently affect overall survival in chemotherapy responders, despite higher intrahepatic recurrence [19]. Together, these studies suggest that a predicted positive margin should no longer be viewed as an absolute contraindication to resection in the modern era.

These observations are further supported in studies examining incident sites and rates of recurrence after R1 resection for CRLM. Andreou and colleagues, in a dual-center cohort of 345 patients, reported that hepatic local recurrence was not detected more frequently after R1 than after R0 resection (*p* = 0.555). Local recurrence itself was not associated with worse overall survival (*p* = 0.436). Yet R1 status still impaired overall survival (*p* = 0.025) [20]. In a per-metastasis analysis of 156 parenchymal R1 resections, Baron and colleagues showed that on-site recurrence at the resection margin occurred in only 31.4 percent of R1 metastases and was isolated (without concurrent recurrence elsewhere) in just 12.8 percent. The development of on-site recurrence did not impact overall survival [21]. Collectively, these observations challenge the historical assumption that R1 margins uniformly produce inferior survival primarily through accelerated and more lethal local recurrence at the resection margin. Instead, they indicate that the prognostic weight of a positive margin is strongly modulated by its precise anatomic location (parenchymal versus vascular) and by underlying tumor biology.

Not all contemporary data support the interpretation of R1 as biologically neutral. Ardito and colleagues, in an analysis of 1428 resection areas in 421 chemotherapy-treated patients, showed that R1 resection remained an independent risk factor for local recurrence even among good chemotherapy responders. Local recurrence at the surgical margin occurred in 24.5 percent of R1 resections versus 8.7 percent of R0 resections (*p* < 0.001), with a clear dose–response relationship between margin width and local recurrence risk [22]. Angelsen and colleagues similarly found that the adverse impact of an R1 margin on survival persisted among chemotherapy-treated patients [23]. A systematic review and meta-analysis by Liu and colleagues encompassing 6790 patients reported that R1 resection was associated with a pooled hazard ratio of 1.60 for five-year overall survival. This association was not attenuated in the modern-chemotherapy subgroup [24]. The contemporary literature therefore remains partially discordant on the overall prognostic weight of R1 as a single category. This seeming discordance can be accounted for by distinguishing R1v versus R1p, which suggests that the adequacy of oncologic margin should instead be defined by violation of Laennec’s capsule.

## 3. The Anatomic and Biological Basis of the R1 Vascular Margin

### 3.1. Laennec’s Capsule and the Surgical Plane

The anatomical foundation for intentional tumor detachment from a major intrahepatic vessel is Laennec’s capsule. This dense fibrous layer can be thought of as encasing the hepatic parenchyma. During embryologic development, this encapsulated liver parenchyma is invaginated by hepatic vein and Glissonian pedicles during organogenesis. First described in the early nineteenth century, Laennec’s capsule was initially discounted to be an erroneous or irrelevant histopathologic discovery, and the notion of a Glissonean capsule enveloping the visceral portion of liver became reigning paradigm of liver anatomy.

More recent cadaveric studies performed by Sugioka et al. have elegantly demonstrated the erroneous nature of Glissonean liver capsule, and have re-demonstrated the existent of a distinct fibrous sheath that excludes hepatic parenchyma from Glissonean pedicles and hepatic veins [25].

Thus, when a tumor abuts but has not invaded a hepatic vein or portal pedicle, the surgeon can enter and develop this plane bluntly. This technique allows the tumor to be peeled off the vessel wall with minimal bleeding. The resulting margin, while microscopically positive to the pathologist’s eye, may now be understood as an oncologically “negative” margin based on respect and preservation of anatomic borders.

### 3.2. Histopathologic Evidence for Detachment

Several lines of histopathologic evidence further support tumor detachment as an oncologically adequate alternative to vascular resection in carefully selected patients. Baumgart and colleagues examined 32 patients who underwent hepatectomy with en-bloc major vessel resection for suspected involvement. Histopathology confirmed true tumor infiltration of the vessel wall in only 6 of 32 cases (19 percent). In all remaining cases the R1 margin was parenchymal, while all vascular wall margins were R0 [26]. Similar findings appear in multiple high-volume series, where preoperative imaging substantially overestimates true vascular invasion. The implication is clear. In a substantial proportion of patients whose tumors appear to abut or invade major vessels on preoperative imaging, the tumor is adherent to but not invading the vessel wall. Meticulous subadventitial dissection along Laennec’s capsule can therefore achieve a complete macroscopic resection without the need for vascular reconstruction.

### 3.3. Biological Rationale for Differential Behavior of R1p and R1v

Although a formal biological explanation for the differential behavior of R1p and R1v remains unclear, several plausible mechanisms have been proposed. Transected hepatic parenchyma after chemotherapy frequently exhibits sinusoidal obstruction, capillarization, and an inflammatory, growth-factor-rich milieu (including hepatocyte growth factor, vascular endothelial growth factor, and platelet-derived growth factor) that may create a permissive niche for microscopic residual disease engraftment and outgrowth. In contrast, the adventitial surface of a major intrahepatic vessel is a dense, collagen-rich, relatively acellular and hypovascular compartment that lacks these regenerative and inflammatory stimuli. This microenvironmental contrast may provide a biologically plausible explanation for the observed difference in local recurrence risk between R1p and R1v margins. Analogous concepts have been explored in primary liver malignancies, where peritumoral stromal characteristics influence recurrence patterns [27]. However, this remains a hypothesis. Direct comparative experimental evidence (e.g., spatial transcriptomics or organoid models of margin-specific interfaces) is currently lacking. The proposed mechanism should therefore be viewed as provisional and hypothesis-generating, and dedicated translational studies will be needed to validate or refute it.

## 4. Differential Oncologic Outcomes of R1 Parenchymal and R1 Vascular Resections

### 4.1. Comparative Survival and Recurrence

The single most important clinical observation supporting the R1p/R1v distinction is that these two categories carry substantially different prognostic weight. In a series of 283 patients with CRLM in contact with major intrahepatic vessels, Liu and colleagues reported five-year overall survival rates of 44.9 percent in the R0 group, 26.3 percent in the R1p group (*p* = 0.009 versus R0), and 34.3 percent in the R1v group (*p* = 0.752 versus R0). On multivariable analysis, parenchymal R1 was an independent predictor of worse overall survival, while vascular R1 was not [7]. This pattern had been foreshadowed by earlier observational work from the same group. Viganò and colleagues, in a cohort of 226 patients, prospectively categorized R1 resections as parenchymal or vascular. They found that local recurrence was more frequent after R1p than after R0, while R1v showed local recurrence rates comparable to R0. On multivariable analysis, R1v (unlike R1p) was not an independent prognostic factor for overall survival [8]. In the twelve-year institutional experience of Torzilli and colleagues, parenchyma-sparing liver resection proved feasible in 146 of 169 patients (86 percent) with tumors contacting major vessels. Tumor–vessel detachment was performed in 66 patients (45 percent). The series reported a mortality rate of 1.4 percent, severe morbidity of 8.2 percent, and a cohort-wide five-year overall survival of 30.7 percent, even though it included anatomically complex and deeply located lesions that would historically have required major hepatectomy [12]. A subsequent analysis by Viganò and colleagues refined these observations further. Among 340 resection areas from 136 patients, local recurrence occurred in 28 of 96 R1par resections compared with only 3 of 31 R1vasc [28] (Table 2).

Subsequent studies have examined how molecular biology influences these outcomes. Procopio and colleagues, in a cohort of 340 patients with known KRAS status, identified a notable interaction between tumor genotype and margin type. In mutated KRAS tumors, local recurrence rates were similar between R0 and R1v and higher after R1p. In wild-type KRAS tumors, R0 showed lower local recurrence than both R1v and R1p [30]. The authors suggested that the biological consequences of vessel-adjacent residual disease depend on tumor genotype, with clear implications for patient selection. Similar molecular sensitivity has been shown with composite risk scores. Wang and colleagues, using the Genetic and Morphological Evaluation (GAME) score, demonstrated that margin clearance was an independent predictor of overall and recurrence-free survival only in patients with low-to-medium GAME scores. In patients with high-risk scores, R0 and R1 resections produced comparable outcomes [31]. Together, these studies highlighted consistent differences in outcomes between R1 parenchymal and R1 vascular margins (Table 3).

### 4.2. Margin Subtype and Local Recurrence

A growing body of refined literature on margin subtype further strengthens the anatomic framework. Ausania and colleagues distinguished R1-contact margins (tumor at the inked cut edge, 0 mm) from R1 margins measuring less than 1 mm but greater than 0 mm. They reported that surgical margin recurrence was significantly higher in the R1-contact group (30.2 percent versus 8.3 percent; *p* = 0.036). On multivariable analysis, the R1-contact margin was the only independent predictor of surgical margin recurrence [29]. This finding parallels the R1p/R1v distinction. Treating the R1 category as binary conflates biologically and anatomically distinct entities whose prognostic implications differ markedly.

Several additional modern cohorts have observed that, even when R1 subtype is not explicitly categorized, recurrence patterns are dominated by intrahepatic disease at sites distant from the resection margin. In a contemporary cohort of 138 patients managed with modern perioperative systemic therapy, Konstantinou and colleagues reported that R1 liver margins did not independently affect overall or disease-free survival. The strongest independent predictors of both R1 resection and poor survival were tumor proximity to major vascular structures and T-stage of the primary tumor [32]. These data support a model in which vessel-adjacent R1 margins more often reflect the technical demands of the operation than any shortfall in oncologic adequacy.

### 4.3. Integration into Parenchyma-Sparing Hepatectomy

The R1p/R1v distinction carries practical importance because it allows parenchyma-sparing hepatectomy to be offered to patients whose tumors would otherwise require major resection. Burlaka and colleagues compared 107 patients with peripherally located metastases to 78 patients with metastases in hard-to-reach central liver sites. They reported that R1v was used in 30.7 percent of central cases versus only 5.6 percent of peripheral cases. Within the hard-to-reach cohort, three-year disease-free survival for R1v versus R0 was 33 percent versus 43 percent (*p* = 0.44) [33]. In a series of 700 patients that included 105 with parenchymal R1 margins, Baron and colleagues found that on-site recurrence occurred in only 31.4 percent of R1 metastases and did not affect overall survival [21]. More recently, Torzilli and colleagues described a longitudinal experience of parenchyma-sparing one-stage hepatectomy for multiple CRLM. Systematic use of R1vasc techniques enabled single-stage resection in patients with multifocal bilobar disease while preserving acceptable long-term survival and high feasibility of repeat hepatectomy for recurrence [13].

Meta-analyses support the broader safety of parenchyma-sparing hepatectomy as an alternative to anatomic major hepatectomy for CRLM. A systematic review by Wang and colleagues found no significant differences in five-year overall survival or disease-free survival between parenchyma-sparing and anatomic resection, despite a modestly higher intrahepatic recurrence rate after parenchyma-sparing procedures [34]. This recurrence pattern is largely salvageable through repeat resection or ablation, both of which benefit from prior parenchymal preservation. Taken together, these findings support an integrated surgical strategy in which selective R1v detachment serves as one component of a parenchyma-sparing philosophy. The goal is to expand operability while maintaining oncologic integrity.

## 5. Intentional R1 Vascular Resection: Patient Selection, Evidence, and Caveats

### 5.1. Tumor Biology and Patient Selection

A central tenet of modern precision surgery for CRLM is that the prognostic impact of margin status depends on underlying tumor biology. Wang and colleagues illustrated this principle clearly through stratification by the GAME score. In patients with low or medium GAME scores, margin clearance was an independent predictor of both overall and recurrence-free survival. In patients with high GAME scores, however, no significant difference appeared between R0 and R1 resections [31]. The implication is straightforward. When tumor biology is dominant, the precise number of millimeters of clearance matters less. When biology is favorable, the margin matters more.

A similar lesson comes from analyses focused on RAS status. A systematic review and meta-analysis by Pikoulis and colleagues examined whether RAS status modulates the oncologic benefit of anatomic versus nonanatomic resection. They concluded that anatomic resection conferred a 40 percent improvement in liver-specific disease-free survival and a 28 percent improvement in overall disease-free survival among patients with mutated RAS tumors, with no such benefit in wild-type patients [35]. A complementary systematic review by Papaconstantinou and colleagues reached consistent conclusions regarding the greater sensitivity of mutated RAS disease to margin status [36]. Together, these data argue against a uniform parenchyma-sparing approach. Instead, they support biology-tailored selection of both resection type and acceptable margin.

BRAF V600E mutation represents an even more aggressive molecular subset that warrants particular attention. In a large multicenter cohort of 849 patients undergoing hepatectomy for CRLM, the presence of BRAF V600E mutation was independently associated with markedly worse overall survival (HR 2.76, 95% CI 1.74–4.37; *p* < 0.001) and disease-free survival (HR 2.04, 95% CI 1.30–3.20; *p* = 0.002), with stronger prognostic impact than KRAS mutation [37]. In patients with liver-limited BRAF V600E disease, hepatectomy has been associated with improved survival compared with systemic therapy alone (median OS 35 vs. 20 months overall; 28 vs. 20 months in propensity-matched analysis) [38], yet absolute outcomes remain poor, with median survival after resection typically in the 30–35 month range and 5-year survival often below 30% in modern series. While specific evidence related to intentional R1v resection in BRAF-mutated tumors is lacking, given its more aggressive natural history, it should likely be approached with even greater caution than the other unfavorable tumor biologies outlined above.

For patients with favorable biology (wild-type KRAS, objective response to systemic therapy, and low GAME score), a planned R1v resection can be offered with reasonable confidence that the oncologic outcome will approximate that of an R0 resection. For patients with adverse biology, particularly mutated KRAS in conjunction with multifocal or large-volume disease, intentional R1v should be used more cautiously. BRAF-mutated disease likely warrants the most conservative stance of all molecular subsets. In these cases, a higher threshold should be applied for wider resection or formal vascular reconstruction.

### 5.2. Evidence for Safety and Efficacy of Intentional R1

Deliberate R1 resection, when undertaken within a multidisciplinary framework, has been shown to be both safe and oncologically acceptable in well-selected patients. Jenvrin and colleagues performed a propensity score-matched case–control analysis of 26 patients in whom an intentional R1 resection was planned preoperatively. These patients were matched 1:4 to 98 R0 controls. After adjustment by inverse probability of treatment weighting, overall and event-free survival did not differ significantly between the intentional R1 and R0 groups (HR 1.19, 95% CI 0.54–2.62 and HR 1.67, 95% CI 0.93–3.03). The authors concluded that intentional R1 resection is an acceptable therapeutic strategy when R0 resection is not feasible [39]. Hosokawa and colleagues demonstrated that long-term cure, defined as five or more years of disease-free interval after the last curative intervention, was achieved in 18 percent of R1 patients. Objective response to ten or fewer cycles of preoperative chemotherapy was the principal independent predictor of cure [17]. Kim and colleagues, using propensity score matching in 368 patients undergoing simultaneous colorectal and liver resection for synchronous CRLM, found that R1 margin status was not independently associated with overall or recurrence-free survival once baseline tumor burden was balanced [40]. Pencovich and colleagues reported similar results. Although R1 resections occurred in patients with more aggressive disease biology and were associated with higher perioperative morbidity, the long-term oncologic outcome of R1 patients was non-inferior to that of R0 patients on multivariable analysis [41] (Table 4).

### 5.3. Caveats and Boundaries of the Approach

These encouraging data must be weighed against several persistent cautions. First, the literature remains discordant on the magnitude of the R1 effect across the overall CRLM population. The meta-analysis by Liu and colleagues, which encompassed 6790 patients, reported a pooled hazard ratio of 1.60 for five-year overall survival after R1 versus R0 resection. This association was not attenuated in the modern-chemotherapy subgroup [24]. A large multicenter experience by Margonis and colleagues similarly found that margin width within the R0 category influenced survival in specific molecular subgroups [42]. These findings suggest that even microscopically negative (R0) margins are associated with inferior outcomes in certain high-risk molecular subsets. Consequently, intentional R1v resection should be approached with particular caution in patients with adverse biology, where the supporting evidence for this technique remains limited. The favorable performance of R1 observed in modern institutional series is therefore not uniformly replicated, and caution is warranted when generalizing results from high-volume, highly specialized centers to routine practice.

Second, the favorable oncologic behavior of R1v is specific to CRLM and does not extend to other hepatic tumors. In intrahepatic cholangiocarcinoma, for example, R1 vascular margins have been associated with markedly inferior disease-free survival compared with R0 and should generally be avoided [43]. Third, achieving comparable outcomes between R1v and R0 requires surgeon expertise with intraoperative ultrasound, familiarity with Laennec’s capsule dissection, and high-volume institutional experience. In less specialized settings an attempt at tumor–vessel detachment may produce a worse outcome than a well-performed major hepatectomy. Fourth, although contemporary single-center experience suggests that in the era of modern perioperative systemic therapy both hepatic recurrence and survival outcomes following hepatectomy for CRLM appear unaffected by the presence of R1 resection margins, tumor proximity to major vessels itself remains an independent predictor of poorer survival. This indicates that the disease biology signaled by vessel proximity cannot be wholly overcome by technique alone [32]. Finally, standardized pathologic reporting of margin subtype—ideally distinguishing R1 vascular, R1 parenchymal, and R1-contact—is required to enable cross-cohort comparison and rigorous validation. Such reporting is currently absent from most synoptic reporting templates [29].

## 6. Technical Considerations and Intraoperative Assessment

### 6.1. Preoperative Imaging and Planning

**Figure 1:**
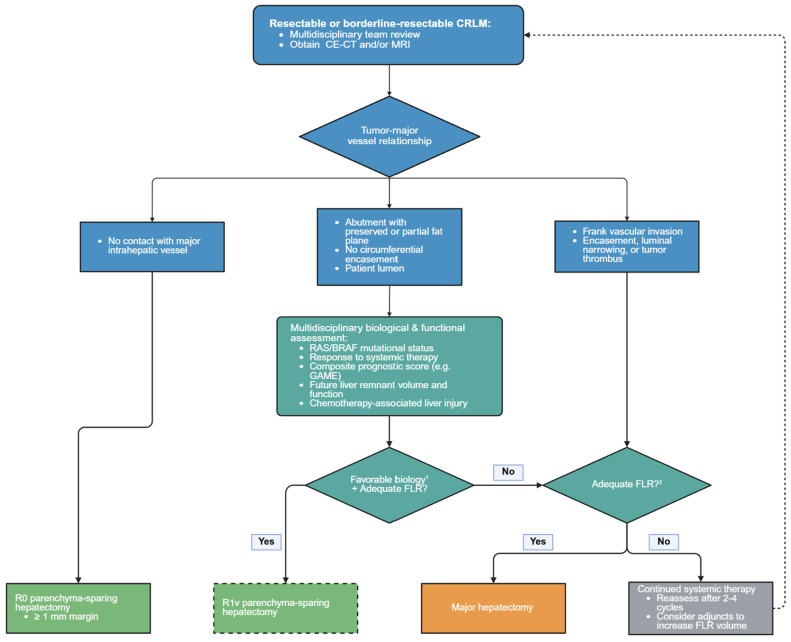
Operative decision algorithm for planned R1 vascular resection in colorectal liver metastasis. ^1^ The distinction between “favorable” and “unfavorable” biology is not binary; the figure depicts a threshold for illustrative purposes. Biological inputs should be weighed together with anatomical and functional factors in multidisciplinary discussion. ^2^ Functional liver remnant thresholds depend on liver quality: typically ≥30% in normal parenchyma and ≥40% in chemotherapy-exposed or steatotic liver; surgeon and institutional judgment applies.

The selection of patients for intentional R1v resection begins with high-quality preoperative imaging. Contrast-enhanced computed tomography or hepatobiliary-contrast magnetic resonance imaging is used to characterize the tumor–vessel interface in detail. Key features include the degree of circumferential vessel contact, the presence or absence of a preserved fat plane, and the integrity of the vessel lumen. Suspected involvement of a major vessel is far less often true histologic invasion than preoperative imaging suggests [26]. Therefore, a tumor that contacts but does not obviously encase or occlude a major hepatic vein, portal branch, or the inferior vena cava remains a candidate for attempted detachment. Volumetric planning of the future liver remnant, together with assessment for steatosis, sinusoidal injury, and regenerative nodules after prolonged chemotherapy, provides essential additional guidance (Figure 1).

### 6.2. Intraoperative Ultrasound

Intraoperative ultrasound is indispensable for the safe execution of R1v resection. It allows the surgeon to confirm the number, size, and location of all metastases, to assess tumor–vessel relationships in real time, to identify communicating veins that preserve venous outflow from segments whose main draining vein is sacrificed, and to define a transection plane that approaches but does not transgress the tumor edge. In an intention-to-treat validation of the Liver Tunnel hepatectomy for deep central tumors involving the major hepatic veins at the caval confluence, Torzilli and colleagues achieved 95 percent technical feasibility, zero 90-day mortality, and a major morbidity rate of only 10 percent in 20 patients treated with IOUS-guided tumor–vessel detachment. Only two cut-edge local recurrences were observed at a median follow-up of 15 months, and both occurred in areas where a macroscopic R1 parenchymal margin had been identified intraoperatively [44]. Procopio and colleagues have since shown that the same principles can be applied safely in a minimally invasive setting. Laparoscopic ultrasound-guided R1v resection at the caval confluence offers a parenchyma-sparing alternative to major hepatectomy [45].

### 6.3. Vessel-Skeletonized Parenchyma-Sparing Techniques

For patients with bilobar disease whose metastases abut multiple major venous or portal structures, vessel-skeletonizing parenchyma-sparing hepatectomy preserves the entire vascular framework of the remnant liver. Umeda and colleagues described a systematic technique in which tumors are detached sequentially from the inflow and outflow structures of the remnant liver, with selective use of intraoperative hepatic vein reconstruction when venous continuity cannot be maintained by communicating veins alone [46]. A complementary approach involves selective reconstruction of critical hepatic veins when detachment would otherwise produce unacceptable congestion of the remnant [47]. These technical options markedly expand the feasibility of single-stage resection for patients with multifocal disease who would otherwise require staged hepatectomy or two-stage procedures with portal vein embolization.

### 6.4. Multicenter Evidence on R1 Determinants

A European multicenter analysis of 3387 consecutive patients who underwent open or laparoscopic liver resection for CRLM identified the independent risk factors for R1 resection. These included nonanatomic or mixed anatomic/nonanatomic resection, the number of lesions, and the size of the largest lesion (with thresholds of 45 mm for open surgery and 30 mm for laparoscopic surgery). Blood loss greater than 350 cc was an independent risk factor for R1 in laparoscopic cases, whereas the Pringle maneuver was independently protective [48]. These findings confirm that technical factors such as blood loss, inflow control, and operative approach influence the margin ultimately achieved. They also provide a quantitative framework for preoperative risk modeling of R1 outcomes.

## 7. Discussion and Future Directions

The contemporary evidence reviewed above supports a reconceptualization of the R1 category in CRLM surgery. The traditional binary R0/R1 framework, while still useful for pathologic reporting, obscures a clinically meaningful distinction. R1 parenchymal margins behave as true adverse events, with higher rates of local recurrence and an independent association with poorer survival. In contrast, R1 vascular margins, when achieved in appropriately selected patients, yield oncologic outcomes indistinguishable from those of R0 resection. This distinction rests on a clear biological foundation: the anatomical difference between the cut surface of hepatic parenchyma and the adventitial surface of a major intrahepatic vessel. It is realized in practice through intraoperative ultrasound-guided dissection along Laennec’s capsule. When incorporated into a broader parenchyma-sparing strategy, planned R1v resection enables single-stage, curative-intent surgery for patients with multifocal or centrally located disease who would otherwise require major hepatectomy, staged operations, or be considered unresectable.

It is important to note that large meta-analyses of unstratified R1 versus R0 resections continue to demonstrate an aggregate survival detriment. Far from diminishing the relevance of the present work, this persistent signal underscores the clinical utility of the R1p/R1v distinction. By demonstrating that the adverse prognostic weight resides predominantly in the R1p subset while R1v outcomes approximate those of R0 in adjusted analyses, the subclassification reconciles the apparently conflicting literature and supplies surgeons with an anatomically grounded, actionable framework. The distinction does not negate the importance of margin status; it refines its interpretation in the current era of precision hepatobiliary surgery and effective systemic therapy. The clinical utility of the R1p/R1v distinction is further shaped by tumor biology. Importantly, tumor biology also influences the very feasibility of achieving a safe R1v margin. Features such as RAS or BRAF mutation status, tumor burden, and response to preoperative chemotherapy affect the likelihood of true vascular wall invasion or perivascular satellitosis versus mere adherence. In patients with aggressive molecular profiles, a clean plane along Laennec’s capsule may not exist or may be incomplete, increasing the risk that attempted detachment results in an unrecognized parenchymal margin violation or residual microscopic disease. In contrast, favorable biology increases the probability that a tumor abutting a major vessel can be cleanly detached without compromising oncologic outcome. The R1p/R1v distinction is therefore most useful when interpreted alongside these biologic factors rather than applied uniformly.

Much of the heterogeneity in reported outcomes across studies stems from differences in case mix and analytic approach. These include the proportion of R1 resections that were vascular versus parenchymal, the degree to which tumor biology and chemotherapy response were balanced through propensity-score or multivariable adjustment, and the depth of multidisciplinary integration with modern systemic therapy and salvage options for recurrence. Where these factors align well, outcomes after R1v approach those of R0. Where they do not, R1 retains its historical prognostic weight.

Several important limitations of the current evidence base warrant acknowledgement. The studies supporting the R1p/R1v distinction are almost exclusively retrospective observational cohorts, often from high-volume hepatobiliary centers, and are therefore subject to selection bias and confounding by indication. No randomized controlled trials exist comparing intentional R1v resection with alternative strategies. While propensity-score matching and multivariable adjustment were employed in several key series, residual confounding cannot be excluded. Furthermore, the favorable outcomes reported in the literature derive overwhelmingly from high-volume centers with extensive experience in intraoperative ultrasound and Laennec’s capsule dissection. The generalizability of these findings to lower-volume settings or surgeons less familiar with these techniques remains uncertain. These limitations should be considered when applying these findings to clinical practice.

The interaction between R1v and systemic therapy is also incompletely characterized. Most series demonstrating comparable outcomes between R1v and R0 resection were conducted in patients receiving modern perioperative chemotherapy, frequently with objective radiographic responses. Data on the safety and efficacy of intentional R1v in chemotherapy-naïve patients, or in those with limited or no response to neoadjuvant therapy, remain limited. In these settings, the underlying tumor biology associated with adverse features may have a greater impact on outcomes, and the risk–benefit balance of accepting a vascular-positive margin versus pursuing wider clearance or vascular reconstruction may be less favorable.

An alternative interpretation of the favorable outcomes observed after R1v resection also merits consideration. It is possible that the ability to achieve a clean macroscopic detachment along Laennec’s capsule primarily serves as a marker of tumors with less invasive biology—lesions that are adherent to but have not transgressed the vessel wall. In this view, the good outcomes attributed to R1v may reflect favorable tumor selection rather than proving that the margin technique itself is oncologically equivalent to R0. While these two interpretations are not mutually exclusive, this possibility underscores the importance of rigorous patient selection and highlights the need for future studies that incorporate preoperative molecular profiling or intraoperative histologic assessment of the detached interface to better separate the effects of surgical technique from inherent tumor biology.

For the practicing hepatobiliary surgeon, several practical conclusions follow. First, a complete macroscopic resection remains the irreducible surgical goal. A negative margin of at least 1 mm should stay the default target whenever it can be achieved without excessive parenchymal sacrifice or unacceptable operative risk. Second, when a tumor abuts a major intrahepatic vessel, preoperative planning should explicitly consider whether an intentional R1v detachment offers a reasonable alternative to vascular resection and reconstruction. Routine use of intraoperative ultrasound is essential to confirm the absence of deep invasion. Third, patient selection for intentional R1v should integrate both conventional prognostic features (number, size, and location of metastases) and biological markers (RAS status, response to chemotherapy, and composite scores such as GAME). Greater caution is warranted in patients with adverse biology. Fourth, the surgical plan should anticipate the likely need for repeat hepatectomy or locoregional salvage of intrahepatic recurrence. The oncologic equivalence of R1v to R0 observed in published series depends in part on the effective treatment of subsequent recurrence.

Several research priorities emerge from this synthesis. First, prospective multicenter registries that employ standardized pathologic reporting of R1 subtype (parenchymal, vascular, and R1-contact) would allow the generalizability of single-center findings to be tested in routine practice. Second, dedicated randomized or quasi-experimental studies comparing intentional R1v resection with alternative strategies (vascular resection with reconstruction, wider parenchymal resection, or systemic therapy alone) in biologically stratified cohorts would provide higher-level evidence for patient selection. Third, molecular and transcriptomic characterization of the parenchymal versus adventitial surface at the time of resection may clarify the biological basis for the differential behavior of R1p and R1v. Such work could also identify patients in whom the adventitial surface is less favorable than current evidence suggests. Fourth, focused study of the BRAF-mutated subgroup and of microsatellite-unstable CRLM in the era of immunotherapy will be needed to extend the R1v framework to these molecularly distinct populations. Fifth, integration of R1v techniques into minimally invasive and robotic platforms should be accompanied by ongoing scrutiny of margin quality. This is particularly important given evidence that broader adoption of minimally invasive hepatectomy can be associated with higher R1 rates in less selected cohorts [46].

## 8. Conclusions

Surgeons have moved away from the rigid pursuit of uniformly wide R0 resection toward a more nuanced, anatomically and biologically informed framework. Within this framework, R1 vascular margins are recognized as oncologically distinct from, and in selected patients equivalent to, traditional R0 margin resection. The evolving data that define oncologically adequate margins in CRLM reflect many of the anatomic and technical insights that have shaped modern liver surgery. While early approaches often required major hepatectomy, advances in technology and technique have enabled more radical yet parenchyma-sparing operations. Grounded in the anatomy of Laennec’s capsule and realized through intraoperative ultrasound-guided parenchyma-sparing hepatectomy, the planned use of R1v expands surgical candidacy for patients with complex CRLM without compromising oncologic principle. Prospective, multicenter data with standardized pathologic reporting will be required to consolidate and generalize this approach. In the meantime, the available evidence supports a central proposition: in CRLM, oncology recapitulates surgical anatomy.

## Figures and Tables

**Table 1:** Evolution of the margin paradigm in colorectal liver metastases: three chronological eras.

Feature	Era I: “1-cm Rule” (Pre-2005) ^1^	Era II: 1 mm Threshold and Biology-as-Surrogate (2005–2015)	Era III: Anatomic Subclassification (2015–Present)
Definition of a “safe” margin	Tumor-free parenchymal margin ≥ 10 mm.	Any microscopically negative margin (≥1 mm); R1 defined as <1 mm; additional clearance beyond 1 mm confers no survival benefit.	Margin subclassified by anatomical location: R0 (≥1 mm); R1p (tumor within 1 mm of transected parenchymal surface); R1v (tumor along adventitia of a major intrahepatic vessel after detachment); R1-contact (0 mm at inked edge).
Dominant surgical approach	Anatomic major hepatectomy with wide parenchymal clearance; tumors abutting major vessels often deemed unresectable or treated with en-bloc vascular resection and reconstruction.	Acceptance of narrower margins; expanded use of perioperative systemic therapy (oxaliplatin- or irinotecan-based regimens ± bevacizumab or anti-EGFR antibodies); R1 “by necessity” increasingly tolerated when R0 not feasible.	Parenchyma-sparing hepatectomy with IOUS-guided tumor–vessel detachment along Laennec’s capsule; intentional R1v as alternative to major hepatectomy or vascular resection; vessel-skeletonizing techniques and selective hepatic vein reconstruction; biology-tailored selection (RAS status, chemotherapy response, GAME score).
Landmark studies	Scheele et al. 1995 [10]; Fong et al. 1999 [1].	Pawlik et al. 2005 [2]; de Haas et al. 2008 [4]; Hamady et al. 2014 [3]; Sadot et al. 2015 [11].	Viganò et al. 2016 [8]; Torzilli et al. 2017 [12]; Liu et al. 2023 [7]; Torzilli et al. 2025 [13].
5-year overall survival (representative figures from landmark cohorts)	Fong 1999 [1]: 37% 5-year OS after resection in 1001 consecutive patients, with positive margin an independent predictor of poor outcome (*p* = 0.004); R1 generally viewed as oncologic failure without durable cure.	de Haas 2008 [4]: R0 61% vs. R1 57% (*p* = 0.27) with perioperative chemotherapy; Sadot 2015 [11] demonstrated a stepwise survival gradient across margin widths, with submillimeter clearance conferring significant advantage over a 0 mm margin.	Torzilli 2017 [12]: R1v 44.5% vs. R1p 25.8%; Liu 2023 [7]: R0 44.9% vs. R1p 26.3% vs. R1v 34.3% (R1v vs. R0 *p* = 0.752).

^1^ Cohort predates modern systemic therapy.

**Table 2:** Contemporary evidence on anatomical subclassification of R1 margins in colorectal liver metastases.

Study (Year) [Ref]	Design	*n*	Margin Definitions	Local Recurrence	Survival	Key Findings
Viganò et al. 2016 [8]	Prospective observational cohort	226 patients/627 resection areas; 46 (20.4%) R1Vasc and 107 (47.3%) R1Par patients	R0: ≥1 mm; R1Par: tumor at transected parenchymal surface; R1Vasc: tumor at adventitia of a major intrahepatic vessel after detachment	Per-patient: R0 5.3%, R1Vasc 4.3% (NS); R1Par 19.6% (*p* < 0.05 vs. R0). Per-resection-area: R0 1.5%, R1Vasc 3.9% (NS); R1Par 13.6% (*p* < 0.05 vs. R0). Hepatic-only recurrence higher in R1Par (49.5% vs. 36.1%, *p* = 0.042)	Mature 5-year OS not reported (median follow-up 33 months)	R1Par was an independent negative prognostic factor for OS on multivariable analysis (*p* = 0.034); R1Vasc was not significantly different from R0 for either local recurrence or OS. First prospective validation of the R1p/R1v distinction
Torzilli et al. 2017 [12]	Single-center 12-year institutional experience; parenchyma-sparing liver resection for CRLM with major vascular contact	169 patients with vascular contact; PSH feasible in 146 (86%); 66 (45%) tumor–vessel detachment, 25 (17%) partial HV resection, 30 (21%) outflow preservation via communicating veins	Same classification as Viganò 2016 [8]	Parenchyma-sparing strategy failed in 14 (7%) due to recurrence in spared parenchyma or cut edge; 13 were radically retreated	Cohort-wide 5-year OS 30.7%; mortality 1.4%; severe morbidity 8.2%	Demonstrated feasibility, safety, and durable oncologic outcomes of parenchyma-sparing hepatectomy with R1Vasc detachment for deeply located CRLM; operationalized R1v within the “radical but conservative” doctrine
Viganò et al. 2022 [28]	Prospective observational cohort stratifying margin status by histopathological growth pattern (HGP)	136 patients/340 resection areas; 143 R0wide, 70 R0min, 31 R1vasc, 96 R1par	R0wide: > 1 mm; R0min: 1 mm; R1vasc: 0 mm with tumor–vessel detachment; R1par: tumor exposure along transection plane	36 local recurrences (11%, median follow-up 21 months): 1 after R0wide, 4 after R0min, 3 after R1vasc, 28 after R1par. In R1par group, local recurrence 29% independent of HGP. In R1vasc and R0min groups, local recurrence higher with replacement HGP (R1vasc: 29% vs. 4%; R0min: 11% vs. 4%)	Not primary endpoint	R1par carries a high, HGP-independent local recurrence risk; R1vasc local recurrence is low except in the replacement-HGP subset. Introduces R0min/R0wide subdivision, reinforcing that margin width beyond 1 mm is prognostically irrelevant in the absence of replacement HGP
Ausania et al. 2022 [29]	Single-center retrospective cohort with granular R1 subclassification	Cohort stratified by depth of R1 positivity (R1-contact vs. R1 < 1 mm)	R1-contact: tumor at inked cut edge (0 mm); R1 < 1 mm: tumor-free margin > 0 and <1 mm	Surgical margin recurrence: 30.2% (R1-contact) vs. 8.3% (R1 < 1 mm); *p* = 0.036	Not primary endpoint	R1-contact margin was the only independent predictor of surgical margin recurrence on multivariable analysis; demonstrates that even within R1 parenchymal margins, depth of positivity carries differential prognostic weight. Complements the R1p/R1v anatomical axis with a granular-depth axis
Liu et al. 2023 [7]	Single-center retrospective cohort restricted to CRLM in contact with major intrahepatic vessels	283 patients	R0: ≥1 mm; R1p: tumor at parenchymal surface; R1v: tumor at vessel adventitia after detachment	Not primary endpoint	5-year OS: R0 44.9%, R1p 26.3%, R1v 34.3% (R1p vs. R0 *p* = 0.009; R1v vs. R0 *p* = 0.752)	On multivariable analysis, R1p was an independent predictor of worse OS; R1v was not. Largest dedicated cohort of vessel-contact CRLM and most rigorous confirmation of the R1p/R1v differential in a non-Milan setting

**Table 3:** Summary of Key Observational Studies Reporting Outcomes Stratified by R1 Parenchymal versus R1 Vascular Margins in Colorectal Liver Metastases.

Study (Year)	Design/*n*	R1 Definitions	Overall Survival	Local Recurrence	Key Adjusted Findings	Notes/Limitations
Viganò et al. 2016 [8]	Prospective observational cohort 226 patients 627 resection areas	R0: ≥1 mm R1Par: tumor at parenchymal surface R1Vasc: tumor at vessel adventitia after detachment	Mature 5-year OS not reported (median follow-up 33 months)	R1Par: 13.6% R1Vasc: 3.9% R0: 1.5% (*p* < 0.05 R1Par vs. R0)	R1Par independent negative predictor of OS (*p* = 0.034); R1Vasc not significantly different from R0	First prospective validation of the R1p/R1v distinction
Torzilli et al. 2017 [12]	Single-center 12-year experience 169 patients with vascular contact; PSH feasible in 146 (86%)	R1Vasc: tumor–vessel detachment along Laennec’s capsule	Cohort-wide 5-year OS: 30.7%	Parenchyma-sparing failed in 7% due to cut-edge or spared parenchyma recurrence	Demonstrated feasibility and safety of parenchyma-sparing hepatectomy with R1Vasc for deeply located CRLM	High-volume single-center experience; operationalized R1v within ‘radical but conservative’ doctrine
Viganò et al. 2022 [28]	Prospective cohort 136 patients 340 resection areas	R0wide: >1 mm; R0min: =1 mm; R1vasc: 0 mm after detachment; R1par: tumor exposure on transection plane	Not primary endpoint	36 local recurrences (11%): R1par 29%, R1vasc 10%, R0min 6%, R0wide 1%	R1par high local recurrence risk independent of HGP; R1vasc risk low except in replacement HGP	Introduces R0min/R0wide subdivision; margin width >1 mm often irrelevant without replacement HGP
Liu et al. 2023 [7]	Single-center retrospective 283 patients with CRLM contacting major intrahepatic vessels	R0: ≥1 mm; R1p: tumor at parenchymal surface; R1v: tumor at vessel adventitia after detachment	R0: 44.9% R1p: 26.3% R1v: 34.3% (R1v vs. R0 *p* = 0.752)	Not primary endpoint	Multivariable: R1p independent predictor of worse OS; R1v was not	Largest dedicated vessel-contact CRLM cohort confirming R1p/R1v differential
Procopio et al. 2020 [30]	Observational cohort with KRAS stratification 340 patients	R1vasc: tumor detachment from vessel adventitia	Not stratified by margin type in primary survival analysis	KRAS-mut: local recurrence similar R0 vs. R1v, higher after R1p KRAS-wt: R0 lower local recurrence than R1v and R1p	Biological consequences of vessel-adjacent residual disease depend on KRAS status	Supports biology-tailored margin strategy; interaction between genotype and margin type

OS = overall survival; HGP = histopathological growth pattern; PSH = parenchyma-sparing hepatectomy. Studies are listed in chronological order. Data extracted from published reports.

**Table 4:** Contemporary evidence supporting intentional or by-necessity R1 resection in colorectal liver metastases.

Study (Year) [Ref]	Design	*n*	Statistical Approach	R1 Definition	Principal R1 vs. R0 Finding	Clinical Implication
***R1 outcomes in the era of modern perioperative systemic therapy***						
Tanaka et al. 2011 [18]	Single-center retrospective cohort stratified by initial resectability and response to prehepatectomy chemotherapy	310	Stratified subgroup analysis	Microscopically positive margin	R0–R1 OS gap abolished in initially unresectable patients who responded to prehepatectomy chemotherapy	Predicted positive margin should not be an absolute contraindication to resection when chemotherapy response is favorable
Ayez et al. 2012 [16]	Single-center retrospective cohort (Erasmus MC, 2000–2008) stratified by neoadjuvant chemotherapy exposure	264; median follow-up 34 months	Subgroup analysis	Tumor-free margin 0 mm	Without neoadjuvant chemotherapy: median OS R0 53 vs. R1 30 months (*p* < 0.001); with neoadjuvant chemotherapy: median OS R0 65 months vs. R1 not reached (*p* = 0.645)	The adverse prognostic signal of R1 is visible only in chemotherapy-naïve patients and attenuates with neoadjuvant systemic therapy
Konstantinou et al. 2025 [32]	Single-center contemporary cohort managed with modern perioperative systemic therapy	138	Multivariable Cox	Tumor-free margin < 1 mm	R1 did not independently affect OS or DFS; strongest independent predictors of R1 and of poor survival were tumor proximity to major vascular structures and T-stage of the primary tumor	In the era of modern perioperative systemic therapy, R1 is driven by vessel-adjacent anatomy rather than oncologic inadequacy
***R1 as a surrogate for tumor biology: evidence from multivariable and propensity-matched analyses***						
Truant et al. 2015 [6]	Single-center retrospective cohort	273	Univariate followed by multivariable Cox	Tumor-free margin < 1 mm	On univariate analysis R1 was associated with inferior 5-year OS, DFS, and PFS; on multivariable analysis independent predictors were tumor number, size, short interval to CRLM, nodal status, rectal primary, and absence of adjuvant chemotherapy—not R1 itself	R1 status is a surrogate for unfavorable tumor biology rather than an independent cause of poor outcome
Pencovich et al. 2019 [41]	Single-center retrospective cohort (Tel Aviv Sourasky, 2006–2016)	202 (161 R0; 41 R1 [20.3%])	Multivariable Cox	Tumor-free margin < 1 mm	R1 associated with more aggressive disease biology (disease progression on chemotherapy 12.1% vs. 5.5%; inferior vena cava involvement 21.9% vs. 8.7%) and higher major morbidity (19.5% vs. 6.8%); on multivariable analysis R1 was not associated with decreased RFS or OS	R1 is the “cost of marginal resection” in biologically aggressive disease, but long-term outcomes after R1 are non-inferior to R0 after adjustment for biology
Andreou et al. 2021 [20]	Dual-center retrospective cohort (Bern + Berlin, 2012–2017)	345 (282 R0; 63 R1 [18%]); median follow-up 34 months	Multivariable Cox and location-specific recurrence analysis	Histologically positive margin	3-year OS R0 71% vs. R1 40% (*p* < 0.0001); however, hepatic local recurrence was independent of R1 status and did not impact OS; 48% of R1 resections occurred because of close proximity to large vessels that could not be sacrificed	Dissenting data point: R1 retains adverse prognostic weight overall, but the mechanism is not lethal local recurrence; vessel proximity is the operative driver of R1
Sakai et al. 2021 [5]	Single-center retrospective cohort (Japanese, 2001–2016)	232	Propensity score matching	Tumor-free margin < 1 mm	Before PSM: R1 had significantly poorer RFS and OS; after PSM: no significant differences in RFS or OS; R1 was associated with higher intrahepatic and early recurrence, while R0 had higher re-resection rate for recurrence	Apparent R0–R1 survival differences disappear once baseline tumor burden is balanced; “R1 resection itself is not a cause of a poor prognosis, but rather a potent indicator of aggressive tumor biology”
Kim et al. 2023 [40]	Retrospective cohort of synchronous CRLM undergoing simultaneous colorectal and liver resection (2006–2017)	368 (304 R0; 64 R1)	Propensity score matching	Tumor at resection line or involved margin	After PSM: no effect of R1 on OS or intrahepatic RFS with or without preoperative chemotherapy	“Tumor biological characteristics, rather than resection margin status, determine long-term prognosis”; aggressive surgical resection should be considered even when R1 is anticipated
***Planned (intentional) R1 as a therapeutic strategy***						
Jenvrin et al. 2023 [39]	Propensity score-matched case–control study of preoperatively planned intentional R1 (iR1)	26 iR1: 98 R0 (1:4 match)	Propensity score matching + IPTW	Intentional R1 planned preoperatively	After IPTW: OS HR 1.19 (95% CI 0.54–2.62); EFS HR 1.67 (95% CI 0.93–3.03); no significant difference between iR1 and R0	Only dedicated study of *planned* rather than by-necessity R1: “intentional R1 resection is an acceptable therapeutic strategy when R0 resection is not feasible”

## Data Availability

No new data were created or analyzed in this study. Data sharing is not applicable to this article.
